# Influência do Sistema Imunológico nas Doenças Cardiovasculares

**DOI:** 10.36660/abc.20230398

**Published:** 2023-07-21

**Authors:** Mariana Gatto, Gustavo Augusto Ferreira Mota, Marina Politi Okoshi

**Affiliations:** 1 Departamento de Clínica Médica Faculdade de Medicina de Botucatu Universidade Estadual Paulista Botucatu SP Brasil Departamento de Clínica Médica – Faculdade de Medicina de Botucatu – Universidade Estadual Paulista (UNESP), Botucatu, SP – Brasil

**Keywords:** Aterosclerose/fisiopatologia, Insuficiência Cardíaca, Hipertensão, Mortalidade, Infarto do Miocárdio, Sistema Imunológico

O sistema imunológico reage a diversos estímulos como corpos estranhos e dano tecidual. A inflamação é a resposta fisiológica inata do organismo às injúrias teciduais desencadeadas por agentes físicos, químicos e biológicos como infecção, trauma, toxina, e necrose tecidual. Uma vez iniciado, o processo somente cessa com a eliminação ou neutralização do agente agressor e a regeneração do tecido danificado.^1^

O processo inflamatório decorre de complexa reação desencadeada por eventos moleculares e bioquímicos que envolvem células imunes, células do tecido conjuntivo, moléculas inflamatórias e vasos sanguíneos.^1 , 2^ Na inflamação aguda, células residentes e células imunes presentes na lesão liberam histaminas, quimiocinas, interleucinas (IL), e fator de necrose tumoral-α (TNF-α) que aumentam a permeabilidade vascular permitindo a infiltração tecidual de fluidos e células imunes como monócitos e neutrófilos. Consequentemente, ocorre a eliminação de patógenos, células necróticas e debris celulares. Finalmente, há inibição das vias inflamatórias e o reparo tecidual ou a substituição do tecido lesado por fibrose.^2^

A amplitude da resposta inflamatória depende do tipo, tempo e intensidade da injúria tecidual. Se o estímulo agressor não for eliminado, o processo inflamatório evolui para fase crônica caracterizada, principalmente, por inflamação aguda persistente e destruição tecidual. Os linfócitos participam ativamente da inflamação crônica, facilitando a sinalização entre vários tipos celulares e a secreção de citocinas inflamatórias.^3^

Doenças cardiovasculares como aterosclerose, insuficiência cardíaca, e hipertensão arterial sistêmica constituem as principais causas de óbito no mundo.^4^ A inflamação participa ativamente da patogênese dessas doenças. Neste Editorial, vamos comentar sobre o envolvimento da inflamação nas doenças cardiovasculares com ênfase em artigos recentemente publicados nos Arquivos Brasileiros de Cardiologia.

A fisiopatologia da aterosclerose é complexa e multifatorial, caracterizada localmente por inflamação crônica, infiltração celular e alterações da coagulação sanguínea. A formação de placas ateroscleróticas na íntima das artérias leva a redução crônica do fluxo sanguíneo. Agudamente, pode ocorrer desestabilidade da placa com ruptura, formação de trombos e oclusão do vaso sanguíneo.^5^

O índice imuno-inflamatório sistêmico (IIS) tem sido avaliado como um biomarcador facilmente acessível e de baixo custo para estratificar a gravidade da doença arterial coronariana.^6 - 8^ O IIS é calculado de acordo com a fórmula: (neutrófilo x plaqueta)/linfócito. Em pacientes com infarto agudo do miocárdio, com ou sem elevação do segmento ST, ou angina, valores elevados de IIS foram associados a maior extensão da lesão aterosclerótica e do dano tecidual, e aumento do tempo de hospitalização.^6^ Adicionalmente, o índice foi preditor independente para eventos adversos maiores em pacientes com infarto agudo e elevação do segmento ST submetidos a intervenção coronária percutânea.^7^ O índice foi particularmente útil quando combinado com tradicionais fatores de risco.^7^

Macrófagos são células especializadas que podem se diferenciar em dois fenótipos. Os macrófagos M1 são células com maior atividade inflamatória e alta capacidade para fagocitose e secreção de mediadores pró-inflamatórios como TNF-α e IL-6.^9^ Ambas as citocinas participam da aterogênese promovendo inflamação vascular, oxidação lipídica, ativação de células endoteliais, proliferação celular, e acúmulo de lipídios por macrófagos.^10^ Por outro lado, os macrófagos M2 possuem menor atividade inflamatória, podendo induzir redução do tamanho de placas ateroscleróticas.^9^ Macrófagos presentes em placas ateroscleróticas acumulam maior quantidade de lipídios quando submetidos a concentração plasmática elevada de lipoproteínas de baixa densidade (LDL) e, neste caso, se diferenciam em células espumosas. O papel dos macrófagos na indução de inflamação em placas ateroscleróticas foi bem ilustrado no estudo de Castro et al.,^11^ A estimulação *in vitro* de macrófagos com LDL oxidado (ox-LDL) aumentou a secreção de IL-6 e TNF-α, que foi proporcional à quantidade de ox-LDL no macrófago.^11^

A ativação imunológica ocorre não somente em placas ateroscleróticas, mas também no miocárdio após infarto agudo ou outras formas de agressão cardíaca. A estimulação do sistema imune, iniciada no miocárdio estruturalmente alterado, se estende para o restante do organismo e pode ser detectada pela presença de marcadores inflamatórios no plasma.^12^

Enquanto a inflamação exacerbada e crônica contribui para pior prognóstico de doenças cardiovasculares,^13^ alguns subtipos de linfócitos, como os TCD4^+^ auxiliares, e mediadores anti-inflamatórios contribuem para reduzir o tamanho e a progressão de placas ateroscleróticas. A IL-10 e o fator de crescimento transformador tipo beta são exemplos de citocinas anti-inflamatórias que atenuam a lesão causada por inflamação; em elevadas concentrações plasmáticas, associam-se a melhor prognóstico da doença arterial coronariana.^14^ Ainda no cenário de mecanismos anti-inflamatórios, a IL-35 é uma citocina com propriedades anti-inflamatórias e imunossupressoras, capaz de suprimir funções deletérias dos linfócitos TCD4+ e induzir diferenciação de células regulatórias. Recentemente, Oflar et al.,^15^ sugeriram que, em pacientes com doença arterial coronariana, baixa concentração plasmática de IL-35 pode estar associada a maior extensão da doença.

Nas últimas décadas, a comunidade científica mostrou que ativação do sistema imunológico participa ativamente da homeostase cardiovascular em situações fisiológicas e patológicas. Entretanto, ainda há necessidade de melhor entendimento sobre o envolvimento da inflamação na fisiopatologia das doenças cardiovasculares para que se possa conduzir terapeuticamente o sistema imunológico para o tratamento de pacientes.

## References

[1] Akhmerov A, Parimon T (2022). Extracellular vesicles, inflammation, and cardiovascular disease. Cells.

[2] Libby P, Loscalzo J, Ridker PM, Farkouh ME, Hsue PY, Fuster V (2018). Inflammation, immunity, and infection in atherothrombosis. J Am Coll Cardiol.

[3] Golia E, Limongelli G, Natale F, Fimiani F, Maddaloni V, Pariggiano I (2014). Inflammation and cardiovascular disease: from pathogenesis to therapeutic target. Curr Atheroscler Rep.

[4] Tsao CW, Aday AW, Almarzooq ZI, Anderson CA, Arora P, Avery CL (2023). Heart disease and stroke statistics—2023 update: a report from the American Heart Association. Circulation.

[5] Pellegrini C, Martelli A, Antonioli L, Fornai M, Blandizzi C, Calderone V (2021). NLRP3 inflammasome in cardiovascular diseases: pathophysiological and pharmacological implications. Med Res Ver.

[6] Gur DO, Efe MM, Alpsoy S, Akyüz A, Uslu N, Çelikkol A (2022). Systemic immune-inflammatory index as a determinant of atherosclerotic burden and high-risk patients with acute coronary syndromes. Arq Bras Cardiol.

[7] Saylik F, Akbulut T (2022). Systemic immune-inflammation index predicts major cardiovascular adverse events in patients with ST-segment elevated myocardial infarction. Arq Bras Cardiol.

[8] Adali MK, Buber I, Sen G, Yilmaz S (2022). Relationship between systemic immune-inflammation index and coronary collateral circulation in patients with chronic total occlusion. Arq Bras Cardiol.

[9] Yang S, Yuan HQ, Hao YM, Ren Z, Qu SL, Liu LS (2020). Macrophage polarization in atherosclerosis. Clin Chim Acta.

[10] Ridker PM, Rane M (2021). Interleukin-6 signaling and anti-interleukin-6 therapeutics in cardiovascular disease. Circ Res.

[11] Castro CA, Buzinari TC, Lino RL, de Araújo HS, Aníbal FF, Verzola RM (2022). Profile of IL-6 and TNF in foam cell formation: an improved method using fluorescein isothiocyanate (FITC) probe. Arq Bras Cardiol.

[12] Martinez PF, Bonomo C, Guizoni DM, Oliveira SA, Damatto RL, Cezar MD (2016). Modulation of MAPK and NF-κB signaling pathways by antioxidant therapy in skeletal muscle of heart failure rats. Cell Physiol Biochem.

[13] Pinheiro LF, Garzon S, Mariani J, Prado GF, Caixeta AM, Almeida BO (2022). Inflammatory phenotype by OCT coronary imaging: specific features among de novo lesions, in-stent neointima, and in-stent neo-atherosclerosis. Arq Bras Cardiol.

[14] Anguera I, Miranda-Guardiola F, Bosch X, Filella X, Sitges M, Marín JL (2002). Elevation of serum levels of the anti-inflammatory cytokine interleukin-10 and decreased risk of coronary events in patients with unstable angina. Am Heart J.

[15] Oflar E, Sahin MH, Demir B, Ertugrul AS, Oztas DM, Beyaz MO (2021). Interleukin-35 levels in patients with stable coronary artery disease. Arq Bras Cardiol.

