# Supplementary material for: Influência do Sistema Imunológico nas Doenças Cardiovasculares
Source: Arq Bras Cardiol. 2023 Jul 21;120(8):e20230398. [Article in Portuguese] doi: 10.36660/abc.20230398 (PMC10464861; doi:10.36660/abc.20230398)
Supplement: Translation

# Translation: Influence of the Immune System on Cardiovascular Disease

**Authors:** Mariana Gatto, Gustavo Augusto Ferreira Mota, Marina Politi Okoshi

**Affiliations:** 1 Brasil Departamento de Clínica Médica – Faculdade de Medicina de Botucatu – Universidade Estadual Paulista (UNESP), Botucatu, SP – Brasil

**Keywords:** Atherosclerosis/physiopathology, Heart Failure, Hipertension, Mortality, Myocardial Infarction, Immune System

The immune system reacts to stimuli such as foreign molecules and tissue damage. Inflammation is an innate physiological response of the organism to tissue injuries triggered by physical, chemical, and biological agents such as infection, trauma, toxin, and tissue necrosis. Once started, the process only ends with the elimination or neutralization of the aggressor agent and the regeneration of the damaged tissue.^1^

The inflammatory process results from complex reactions triggered by molecular and biochemical events involving immune cells, connective tissue cells, inflammatory molecules, and blood vessels.^1 , 2^ In acute inflammation, resident cells and immune cells present in the injured tissue release histamines, chemokines, interleukins (IL), and tumor necrosis factor-α (TNF-α) that increase vascular permeability allowing tissue infiltration of fluids and immune cells such as monocytes and neutrophils. Consequently, pathogens, necrotic cells, and cellular debris are eliminated. Finally, inflammatory pathways are inhibited with tissue repair or replacement by fibrosis.^2^

The amplitude of the inflammatory response depends on the type, duration, and intensity of tissue injury. If the aggressor stimulus is not eliminated, the inflammatory process evolves into a chronic phase characterized by persistent acute inflammation and tissue destruction. Lymphocytes participate in chronic inflammation, facilitating signaling between different cell types and the secretion of inflammatory cytokines.^3^

Cardiovascular diseases such as atherosclerosis, heart failure, and systemic arterial hypertension are the main causes of death worldwide.^4^ Inflammation actively participates in the pathogenesis of these diseases. In this Editorial, we will comment on the involvement of inflammation in cardiovascular disease, emphasizing articles recently published in the *Arquivos Brasileiros de Cardiologia* .

The pathophysiology of atherosclerosis is complex and multifactorial, characterized by chronic inflammation, cell infiltration, and blood clotting disorders. Atherosclerotic plaques in the intima of arteries lead to a chronic reduction in blood flow. Acutely, plaque instability may occur with rupture, thrombus formation, and blood vessel occlusion.^5^

The systemic immunoinflammatory index (SII) has been evaluated as an easily accessible and low-cost biomarker to stratify the severity of coronary artery disease.^6 - 8^ The SII is calculated using the formula: (neutrophil x platelet)/lymphocyte. Elevated SII values were associated with a greater extent of atherosclerotic lesions, higher tissue damage, and increased length of hospital stay in patients with acute myocardial infarction or angina.^6^ Additionally, the index was an independent predictor of major adverse events in patients with acute myocardial infarction and ST-segment elevation undergoing percutaneous coronary intervention.^7^ The index was particularly useful when combined with traditional risk factors.^7^

Macrophages are specialized cells that can differentiate into two phenotypes. M1 macrophages have high inflammatory activity with elevated capacity for phagocytosis and secretion of pro-inflammatory mediators such as TNF-α and IL-6.^9^ Both cytokines participate in atherogenesis inducing vascular inflammation, lipid oxidation, endothelial cell activation, cell proliferation, and macrophage lipid accumulation.^10^ On the other hand, M2 macrophages have poor inflammatory activity and may induce a reduction in atherosclerotic plaque size.^9^ Macrophages present in atherosclerotic plaques accumulate a great amount of lipids when subjected to high levels of plasma low-density lipoproteins (LDL); in this case, these cells differentiate into foam cells. The role of macrophages in inducing inflammation in atherosclerotic plaques was well illustrated in the study by Castro et al.^11^ *In vitro* stimulation of macrophages with oxidized LDL (ox-LDL) increased IL-6 and TNF-α secretion, which was proportional to the amount of ox-LDL in the macrophage.^11^

Immunologic activation occurs not only in atherosclerotic plaques but also in the myocardium after acute infarction or other types of cardiac injury. The stimulation of the immune system, initiated in the structurally altered myocardium, spreads to the bloodstream and can be detected by increased levels of plasma inflammatory biomarkers.^12^

While exacerbated and chronic inflammation contributes to a worse prognosis in cardiovascular disease,^13^ some lymphocyte subtypes, such as TCD4+ helper and anti-inflammatory mediators, reduce the size and progression of atherosclerotic plaques. IL-10 and transforming growth factor beta are examples of anti-inflammatory cytokines that attenuate damage caused by inflammation; in high plasma concentrations, they are related to a better prognosis of coronary artery disease.^14^ Still, in the scenario of anti-inflammatory mechanisms, IL-35 is a cytokine with anti-inflammatory and immunosuppressive properties that can suppress the deleterious function of TCD4+ lymphocytes and induce differentiation of regulatory cells. Recently, Oflar et al.^15^ suggested that low levels of IL-35 plasma concentration are associated with a greater extension of coronary artery disease.

In recent decades, extensive research has shown that activation of the immunologic system is involved in cardiovascular homeostasis in physiological and pathophysiological conditions. However, a better understanding of the role of inflammation in cardiovascular disease is needed so that immunomodulation of the immune system can be used to treat patients.
